# Clinical and genomic characterization of KRAS G13X non-small cell lung carcinoma

**DOI:** 10.3389/fonc.2026.1825959

**Published:** 2026-09-03

**Authors:** Benjamin J. Staum, Rachaita Lakra, Aditya Ravindra, Sarah L Mott, Deqin Ma, William Zeitler, Margaret Byrne, Nirmal Choradia, Abdul Rafeh Naqash, Raid Aljumaily, Muhammad Furqan

**Affiliations:** 1 HealthPartners Cancer Center at Regions Hospital, Saint Paul, MN, United States; 2 Division of Hematology-Oncology, Dept. of Internal Medicine - University of Iowa Health Care, Iowa City, IA, United States; 3 Holden Comprehensive Cancer Center, University of Iowa Health Care, Iowa City, IA, United States; 4 Molecular Pathology, Dept. of Pathology, University of Iowa Health Care, Iowa City, IA, United States; 5 University of Oklahoma, Stephenson Cancer Center – OU Health, Oklahoma City, OK, United States

**Keywords:** clinical characterization, co-mutations, KRAS G13, molecular characterization, NSCLC

## Abstract

**Background:**

*KRAS* mutations (KRAS^m^)represent the most common oncogenic drivers in non–small cell lung cancer (NSCLC). However, molecular features and clinical implications of the less common, codon 13 mutations (*KRAS* G13X) remain poorly characterized. We conducted a retrospective study to characterize *KRAS* G13X-positive NSCLC.

**Methods:**

We reviewed patients’ charts with KRAS^m^ NSCLC treated at University of Iowa Health Care. Based on intent of therapy, patients were assigned to curative-intent (CI) or palliative-intent (PI) cohorts. Clinicopathologic data, co-mutation profiles, and outcomes were assessed.

**Results:**

A total of 108 CI and 100 PI patients were included. *KRAS* G13X accounted for ~10% of KRAS^m^ NSCLC. *KRAS* G13X tumors were enriched for *STK11*, *KEAP1*, *NFE2L2*, and *TSC1/2* co-mutations. In the CI cohort, *KRAS* G13X had the lowest 12-month disease-free survival (DFS) (41%), though *KRAS* subtype was not independently prognostic. Poor performance status, stage III disease, delayed treatment initiation, and not having *ATM* co-mutations were associated with inferior DFS. *TET2* alterations were associated with worse overall survival (OS). In the PI cohort, *KRAS* G13X had the lowest 12-month progression-free survival (PFS) (17%) and OS (27%) compared to other *KRAS* alterations. On multivariable analysis, *STK11*, *TERT*, and *TSC1/2* alterations remained independently associated with worse survival, while *KRAS* G13X and G12D tended to exhibit poorer OS.

**Conclusions:**

*KRAS* G13X mutations represent a small but clinically meaningful subset of NSCLC defined by a distinctive co-mutation pattern. Outcomes appear driven predominantly by co-mutations. Combinational therapeutic strategies addressing these vulnerabilities can help improve outcomes.

## 1 Introduction

*KRAS* mutations occur in approximately 25–30% of non–small cell lung cancer (NSCLC) in Western populations. *KRAS* encodes a small GTP-binding protein that regulates downstream signaling through the MAPK/ERK, PI3K/AKT, and Ral-GDS pathways. Missense mutations most frequently at codons 12, 13, and 61 impair intrinsic GTPase activity or disrupt interactions with GTPase-activating proteins, resulting in constitutive pathway activation (1–3). Distinct amino-acid substitutions produce unique biochemical and phenotypic profiles. For example, G12C and G12V preferentially activate RAF and Ral-GDS pathways, whereas G12D enhances PI3K signaling (4–6). These differences may underscore allele-specific therapeutic vulnerabilities, as illustrated by the selective activity of sotorasib and adagrasib for *KRAS* G12C and zoldonrasib for *KRAS* G12D NSCLC.

Genomic co-alterations are major determinants of clinical behavior in *KRAS* mutant NSCLC. Co-mutations in *TP53*, *STK11*, and *KEAP1* define biologically distinct subsets with variable immune phenotypes and treatment outcomes (7–9). Published data abundantly characterize the molecular landscape of *KRAS* G12 variants, however clinic-pathologic features of less common codon 13 alterations (*KRAS* G13X), remain poorly described. Prior studies have characterized allele-specific biology (10) and reported clinical outcomes across *KRAS* subtypes (11–15), but dedicated analyses of *KRAS* G13X are limited.

To address this gap, we conducted a single-center retrospective study to characterize the incidence, co-mutational landscape, and clinical outcomes of *KRAS* G13X NSCLC compared with other *KRAS* alleles across curative-intent and palliative-intent treatment settings.

## 2 Methods

### 2.1 Study design

We retrospectively reviewed electronic medical records of adult patients with *KRAS*-mutant NSCLC who received care at the University of Iowa Health Care – Holden Comprehensive Cancer Center and had next-generation sequencing (NGS) performed on tumor tissue samples. Patients were included only if their treatment and follow-up records were available. Patients were assigned to curative-intent (CI) or palliative-intent (PI) cohorts based on clinical stage and treatment approach. We included patients who were diagnosed between January 2016 and Dec 20024. Patients initially treated with curative intent therapy and later developed metastatic disease were eligible for inclusion in the PI cohort.

We recorded variables including age, gender, date of diagnosis, intent of treatment (curative or palliative), Eastern Cooperative Oncology Group (ECOG) performance status, smoking status, histology, sites of metastatic disease where applicable, PD-L1 expression, co-mutations, receipt of therapy, and outcomes.

PD-L1 expression was evaluated by immunohistochemistry with 22C3 (Dako, Carpinteria, CA) or E1L3N (Dako Autostainer Link 48) assays. Somatic alterations including substitutions, small deletions and insertions, and copy number changes was performed using Ampliseq-based next generation sequencing (NGS) with a 213 gene custom-designed panel on a NextSeq 2000 (Illumina Inc., San Diego, CA). Evaluation for gene rearrangements was performed by testing fusion transcripts using the ArcherDx FusionPlex Comprehensive Thyroid and Lung Panel (Integrated DNA Technologies, San Diego, CA) or a custom-designed, laboratory-validated targeted FusionPlex panel. All testing was performed at the Clinical Laboratory Improvement Amendments-certified molecular pathology laboratory at the University of Iowa.

### 2.2 Statistical analysis

Statistical analyses were separately conducted for both cohorts. For the CI cohort, time was calculated from treatment initiation to recurrence or death due to any cause for disease-free survival (DFS). Otherwise, patients were censored at last disease evaluation. In addition, time was calculated from treatment initiation to death due to any cause for overall survival (OS). Otherwise, patients were censored at last known alive. Stage heavily influences treatment selection. Given the limited number of events, only stage was included in the multivariable models to directly adjust for disease extent and indirectly adjust for treatment. For the PI cohort, time was calculated from treatment initiation to progression or death due to any cause for progression-free survival (PFS). Otherwise, patients were censored at last disease evaluation. Time was calculated from treatment initiation to death due to any cause for OS. Otherwise, patients were censored at the last known alive. Survival probabilities were estimated and plotted using the Kaplan-Meier method. Outcomes (DFS, PFS, and OS) of patients with *KRAS* G12C, G12V and non-G12/G13 were combined due to similarity and small sample size of non-G12/G13 cohorts for the Kaplan-Meier curves. Cox regression models were utilized to estimate the effect of patient, clinicopathologic, genetic, and treatment characteristics on DFS, PFS and OS. Multivariable models were built by prioritizing inclusion of mutations associated with each endpoint (DFS, PFS, OS) while adjusting for known or observed associations between clinicopathologic and treatment characteristics and each endpoint as the data allowed (i.e. without overfitting the data). All tests were two-sided and assessed for significance at the 5% level using SAS v9.4 (SAS Institute, Cary, NC). Ethical approval for the study was obtained from the University of Iowa Institutional Review Board (Hawk IRB #202408002).

## 3 Results

### 3.1 Curative intent cohort

A total of 108 patients with *KRAS* mutations were included in this cohort. The majority were female (61%), former smokers (57%) and had stage I-II disease (64%, **Table 1**). Ten patients (8%) harbored *KRAS* G13C/D/R/V alterations (*KRAS* G13X). Eight (7%) patients had alterations in codons E49, Q61 and A146 and are referred to as *KRAS* non-G12/G13 in this manuscript. Most patients with stage I-II disease received surgery (72%) without chemotherapy (91%) or radiation (71%) whereas stage III disease received chemotherapy (79%) and radiation (74%) without surgery (72%). Patients with mutations in codons other than 12 tend to be younger, with median ages of 64 years for *KRAS* G13X and 64 years in patients with *KRAS* mutations in codons other than 12 & 13. Eight patients were never smokers, all harboring alterations on codon 12. Patients with *KRAS* G13X and non-G12/G13 were all either current or former smokers.

**Table 1:** Demographics and clinical characteristics of patients in CI cohort.

Covariate	Level	Total N=108 (%)	*KRAS* variants					
			G12C N=40 (%)	G12V N=22 (%)	G12D N=22 (%)	G12A/F/R/S N=6 (%)	G13X* N=10 (%)	Non-G12/G13^#^ N=8 (%)
Age at Diagnosis	Median	67	68	67	67	70	64	64
	(Min-Max)	(46-85)	(46-81)	(47-84)	(48-79)	(56-84)	(55-85)	(55-76)
Sex	Female	66 (61)	22 (55)	17 (77)	13 (59)	6 (100)	6 (60)	2 (25)
	Male	42 (39)	18 (45)	5 (23)	9 (41)	0 (0)	4 (40)	6 (75)
ECOG PS	0	44 (41)	18 (45)	7 (32)	8 (36)	5 (83)	3 (30)	3 (38)
	1	51 (47)	16 (40)	12 (55)	12 (55)	1 (17)	6 (60)	4 (50)
	2	13 (12)	6 (15)	3 (14)	2 (9)	0 (0)	1 (10)	1 (13)
Smoking Status	Never	8 (7)	2 (5)	2 (9)	3 (14)	1 (17)	0 (0)	0 (0)
	Former	61 (57)	23 (59)	9 (41)	13 (59)	4 (67)	6 (60)	6 (75)
	Current	38 (36)	14 (36)	11 (50)	6 (27)	1 (17)	4 (40)	2 (25)
PD-L1 TPS	0%	47 (44)	16 (40)	9 (41)	12 (55)	3 (60)	5 (50)	2 (25)
	1-100%	60 (56)	24 (60)	13 (59)	10 (45)	2 (40)	5 (50)	6 (75)
Adenocarcinoma	No	19 (18)	8 (20)	2 (9)	5 (23)	0 (0)	3 (30)	1 (14)
	Yes	88 (82)	32 (80)	20 (91)	17 (77)	6 (100)	7 (70)	6 (86)
Stage	I-II	69 (64)	26 (65)	16 (73)	14 (64)	4 (67)	8 (80)	1 (13)
	III	39 (36)	14 (35)	6 (27)	8 (36)	2 (33)	2 (20)	7 (88)
Surgery	No	47 (44)	16 (40)	6 (27)	12 (55)	1 (17)	5 (50)	7 (88)
	Yes	61 (56)	24 (60)	16 (73)	10 (45)	5 (83)	5 (50)	1 (13)
Chemotherapy	No	71 (66)	25 (63)	17 (77)	15 (68)	6 (100)	6 (60)	2 (25)
	Yes	37 (34)	15 (38)	5 (23)	7 (32)	0 (0)	4 (40)	6 (75)
Radiation	No	59 (55)	24 (60)	15 (68)	10 (45)	5 (83)	4 (40)	1 (13)
	Yes	49 (45)	16 (40)	7 (32)	12 (55)	1 (17)	6 (60)	7 (88)
Months From Diagnosis to Treatment	Median	1.1	0.9	0.9	1.1	2.3	1.4	2.0
	(Min-Max)	(0.0-8.0)	(0.0-3.1)	(0.0-4.0)	(0.0-2.8)	(0.0-5.3)	(0.7-7.9)	(0.4-8.0)

*KRAS G13X included patients with *KRAS* G13C (n=3), G13D (n=5) and G13V (n=2).

**^#^***KRAS* non-G12/G13 included *KRAS* E49Q (n=1), Q61A (n=1), Q61H (n=4), Q61L (n=1) and A146S (n=1).

**Figure 1:**
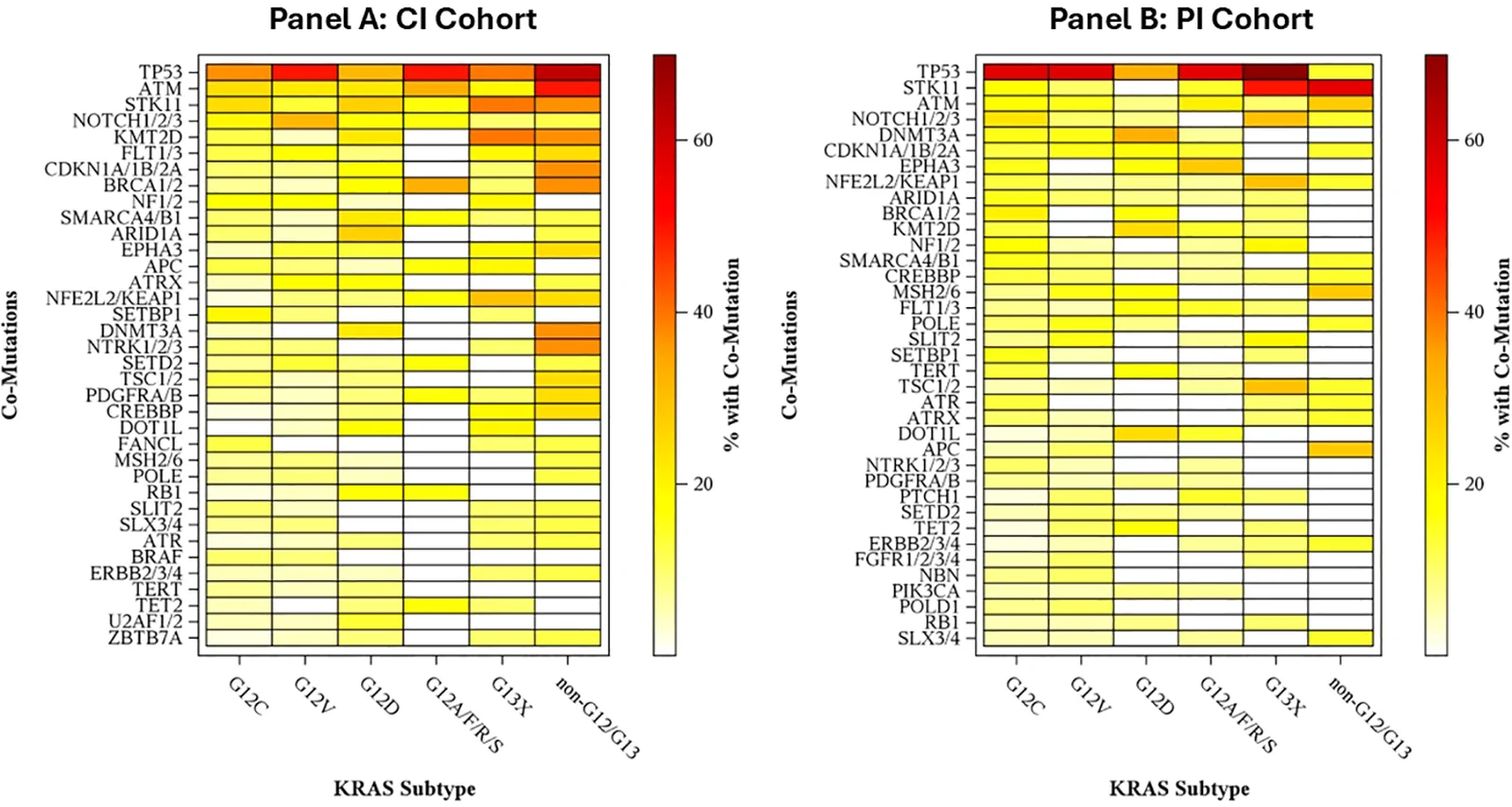
Frequency of co-mutations (y-axis) across various *KRAS* mutated (x-axis) NSCLC. Panel **(A)** represents CI cohort; Panel **(B)** represents PI cohort.

PD-L1 expression did not differ substantially across *KRAS* subtypes, although NSCLC with *KRAS* non-G12/G13 (75%) and *KRAS* G12C (60%) were more frequently PD-L1–positive. Among patients with *KRAS* G13X, 80% presented with stage I–II disease, but only 50% underwent surgery. *TP53* was the most frequently co-mutated gene across all *KRAS* subtypes, followed by *STK11* (**Figure 1A**). *STK11* alterations were more common among *KRAS* G13X and *KRAS* non-G12/G13. Co-mutations in *KMT2D*, *KEAP1*and *NFE2L2* were also more frequent in *KRAS* G13X.

**Figure 2:**
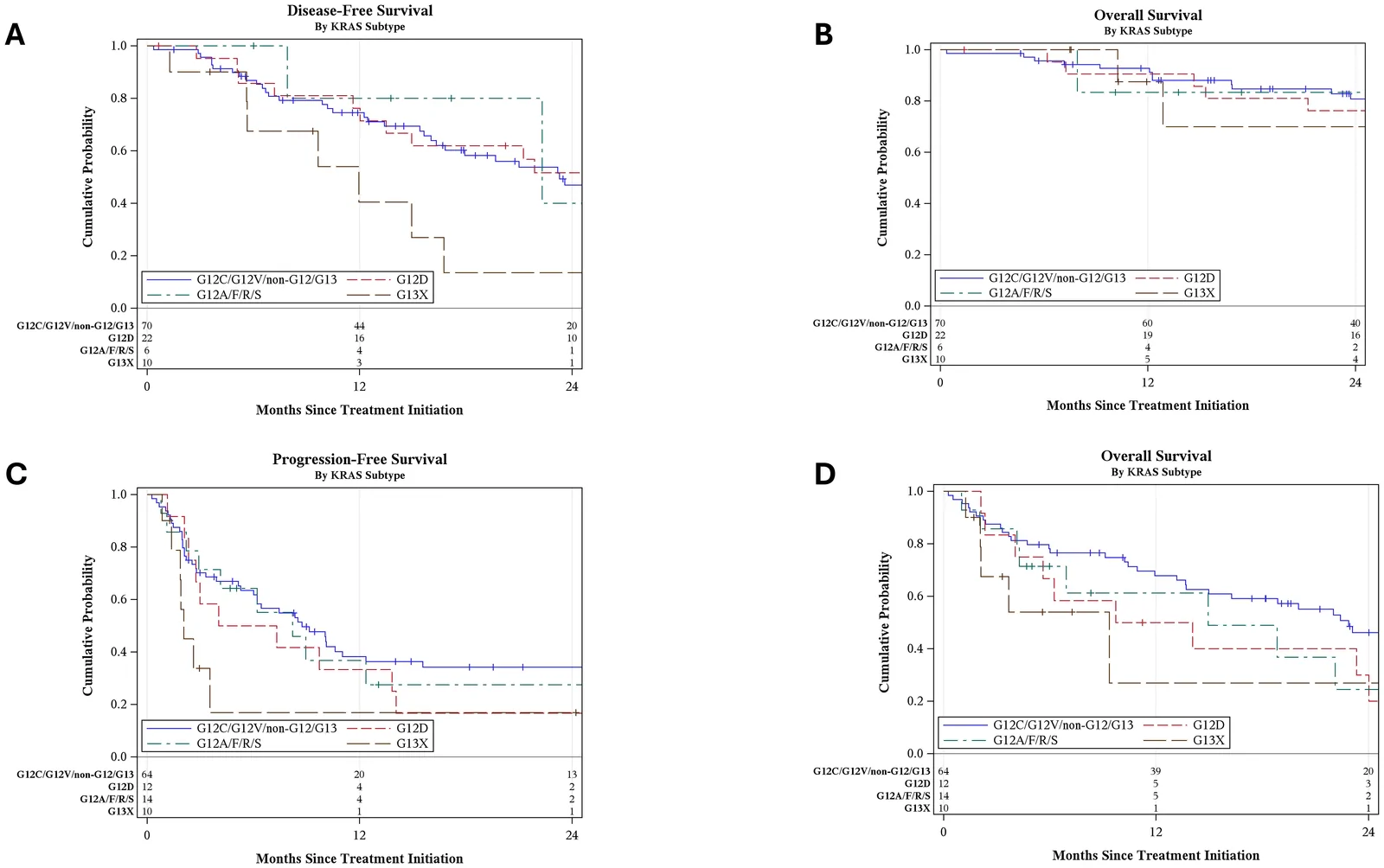
Disease-free, progression-free and overall survival of *KRAS* mutated NSCLC. Kaplan-Meier estimates of DFS **(A)**, OS **(B)** of patients in CI cohort. Kaplan-Meier estimates of PFS **(C)** and OS **(D)** of patients in PI cohort.

At a median follow-up of 27.1 months, 39 patients had died. The. The median DFS was 22.3 months (95% CI: 16.3-27.6 months). The 12-month DFS rates for *KRAS* G12C, G12V, G12D, G12A/F/R/S, G13X and non-G12/G13 were 80% (95% CI: 63-89%), 67% (95% CI: 43-83%), 76% (95% CI: 52-89%), 80% (95% CI: 20-97%), 41% (95% CI: 10-70%) and 71% (95% CI: 26-92%) respectively (**Figure 2A**; **Supplementary Table 1**). On univariate analysis, factors associated with poorer DFS included ECOG PS 2, PD-L1-positivity, stage III disease, not undergoing surgery, receipt of radiation, and longer time to therapy initiation (**Supplementary Table 2**). However, patients with *ATM* co-mutations had better DFS. ECOG PS 2 (HR: 3.01; 95% CI: 1.52-5.95), not having *ATM* co-mutations (HR: 0.53; 95% CI: 0.29-0.95), and increasing months to therapy initiation (HR: 1.24; 95% CI: 1.04-1.47) remained significantly associated with DFS while stage and PD-LI were no longer statistically significant on multivariable analysis (**Table 2**).

**Table 2:** Multivariable models for DFS and OS in the CI Cohort.

Covariate	Level	Disease-free survival				Overall survival			
		Hazard ratio	95% CI		P-value	Hazard ratio	95% CI		P-value
ECOG	2	3.01	1.52	5.95	**<.01**	4.95	2.31	10.62	**<.01**
	0-1	Ref	-	-		Ref	-	-	
Stage	III	1.58	0.91	2.73	0.11	1.15	0.59	2.23	0.69
	I-II	Ref	-	-		Ref	-	-	
PD-L1 TPS	0%	0.56	0.32	1.01	0.05				
	1-100%	Ref	-	-					
*ATM* mutations	Yes	0.53	0.29	0.95	**0.03**				
	No	Ref	-	-					
*TET2* mutations	Yes					3.93	1.30	11.84	**0.02**
	No					Ref	-	-	
Months From Diagnosis to Treatment	Units=1	1.24	1.04	1.47	**0.01**				

p-values in bold indicate statistical significance.

Median OS was 51.8 months (95% CI: 34.7-76.0 months). The 12-month OS rates for *KRAS* G12C, G12V, G12D, G12A/F/R/S, G13X and non-G12/G13 were 92% (95% CI: 78-97%), 91% (95% CI: 68-98%), 90% (95% CI: 67-98%), 83% (95% CI: 27-97%), 88% (95% CI: 39-98%) and 100% respectively (**Figure 2B**; **Supplementary Table 1**). On univariate analysis, ECOG PS 2, not undergoing surgery, receipt of radiation, and having *TET2* alterations were associated with inferior survival (**Supplementary Table 3**). On multivariable analysis, poor performance status (HR: 4.95; 95% CI: 2.31-10.62) and *TET2* co-mutations (HR: 3.93; 95% CI: 1.30-11.84) retained their independent negative prognostic significance (**Table 2**).

### 3.2 Palliative intent cohort

A total of 100 patients with *KRAS* mutations were included in this cohort, the median age was 68 and the majority were female (54%) and former smokers (59%, **Table 3**). Like CI cohort, patients with alterations in *KRAS* non-G12/G13 were more likely to be men. Patients with *KRAS* G13X tended to be relatively younger. First-line treatment for many of the patients was a combination of chemotherapy and immunotherapy (62%), while 19% and 13% of patients received immunotherapy alone and chemotherapy alone respectively. A few patients with *KRAS* G12C received either sotorasib or adagrasib in the first line setting as an off-label therapy.

**Table 3:** Demographics and clinical characteristics of patients in PI cohort.

Covariate	Level	Total N=100 (%)	*KRAS* variants					
			G12C N=38 (%)	G12V N=19 (%)	G12D N=12 (%)	G12A/F/R/S N=14 (%)	G13X* N=10 (%)	Non-G12/G13^#^ N=7 (%)
Age at Diagnosis	Median	68	69	69	71	69	64	72
	(Min-Max)	(42-89)	(42-82)	(48-89)	(50-83)	(57-85)	(45-68)	(49-76)
Sex	Female	54 (54)	21 (55)	14 (74)	4 (33)	9 (64)	5 (50)	1 (14)
	Male	46 (46)	17 (45)	5 (26)	8 (67)	5 (36)	5 (50)	6 (86)
ECOG PS	0	14 (14)	6 (16)	4 (21)	0 (0)	1 (7)	2 (20)	1 (14)
	1	69 (69)	28 (74)	12 (63)	9 (75)	10 (71)	6 (60)	4 (57)
	2	15 (15)	4 (11)	2 (11)	2 (17)	3 (21)	2 (20)	2 (29)
	3	2 (2)	0 (0)	1 (5)	1 (8)	0 (0)	0 (0)	0 (0)
Smoking Status	Never	7 (7)	1 (3)	1 (5)	2 (17)	2 (14)	1 (10)	0 (0)
	Former	59 (59)	21 (55)	10 (53)	9 (75)	8 (57)	7 (70)	4 (57)
	Current	34 (34)	16 (42)	8 (42)	1 (8)	4 (29)	2 (20)	3 (43)
PD-L1 TPS	0%	23 (23)	8 (21)	4 (21)	1 (8)	4 (31)	3 (33)	3 (43)
	1-100%	75 (77)	30 (79)	15 (79)	11 (92)	9 (69)	6 (67)	4 (57)
Adenocarcinoma	No	25 (25)	11 (29)	3 (16)	4 (33)	4 (29)	3 (30)	0 (0)
	Yes	75 (75)	27 (71)	16 (84)	8 (67)	10 (71)	7 (70)	7 (100)
Group	Newly Diagnosed	65 (65)	25 (66)	9 (47)	6 (50)	12 (86)	9 (90)	4 (57)
	Recurrent	35 (35)	13 (34)	10 (53)	6 (50)	2 (14)	1 (10)	3 (43)
Chest metastases (M1a)	No	41 (41)	17 (45)	7 (39)	3 (25)	6 (43)	5 (50)	3 (43)
	Yes	58 (59)	21 (55)	11 (61)	9 (75)	8 (57)	5 (50)	4 (57)
Bone	No	64 (65)	24 (63)	13 (72)	10 (83)	9 (64)	5 (50)	3 (43)
	Yes	35 (35)	14 (37)	5 (28)	2 (17)	5 (36)	5 (50)	4 (57)
Brain	No	64 (65)	20 (53)	17 (94)	9 (75)	8 (57)	6 (60)	4 (57)
	Yes	35 (35)	18 (47)	1 (6)	3 (25)	6 (43)	4 (40)	3 (43)
Liver	No	92 (93)	35 (92)	17 (94)	12 (100)	12 (86)	9 (90)	7 (100)
	Yes	7 (7)	3 (8)	1 (6)	0 (0)	2 (14)	1 (10)	0 (0)
Adrenal	No	87 (88)	35 (92)	16 (89)	11 (92)	12 (86)	8 (80)	5 (71)
	Yes	12 (12)	3 (8)	2 (11)	1 (8)	2 (14)	2 (20)	2 (29)
Muscle/Soft Tissue	No	89 (90)	34 (89)	16 (89)	11 (92)	13 (93)	9 (90)	6 (86)
	Yes	10 (10)	4 (11)	2 (11)	1 (8)	1 (7)	1 (10)	1 (14)
Initial Treatment	Chemo + IO	62 (62)	22 (58)	11 (58)	6 (50)	9 (64)	8 (80)	6 (86)
	Chemo Only	13 (13)	3 (8)	4 (21)	3 (25)	1 (7)	2 (20)	0 (0)
	IO Only	19 (19)	7 (18)	4 (21)	3 (25)	4 (29)	0 (0)	1 (14)
	Targeted Therapy	6 (6)	6 (16)	0 (0)	0 (0)	0 (0)	0 (0)	0 (0)
Months From Diagnosis to Treatment	Median	1.0	1.1	0.7	1.1	1.1	0.7	1.2
	(Min-Max)	(0.0-73.1)	(0.0-7.5)	(0.0-3.2)	(0.4-2.5)	(0.1-73.1)	(0.2-2.9)	(0.3-5.4)

*KRAS G13X included patients with *KRAS* G13C (n=3), G13D (n=5), G13R (n=1) and G13V (n=1).

**^#^***KRAS* non-G12/G13 included *KRAS* Q61H (n=6) and Q61L (n=1).

*TP53* remained the most frequent co-mutated gene, followed by *STK11*, which occurred more commonly in *KRAS* G13X and non-G12/G13 subtypes. *KEAP1*, *NFE2L2*, *NOTCH1/2/3* and *TSC1/2* alterations were enriched in *KRAS* G13X tumors (**Figure 1B**).

At a median follow-up of 12.6 months, 57 patients had died. The median PFS was 8.2 months (95% CI: 4.1-10.1 months). The 12-month PFS rates for *KRAS* G12C, G12V, G12D, G12A/F/R/S, G13X and non-G12/G13 were 43% (95% CI: 26-59%), 33% (95% CI: 13-55%), 33% (95% CI: 10-59%), and 37% (95% CI: 12-62%), 17% (95% CI: 1-50%) and 29% (95% CI: 4-61%) respectively (**Figure 2C**; **Supplementary Table 1**). On univariate analysis, poorly differentiated or squamous histology, presence of bone metastases, adrenal metastases, soft-tissue metastases, *STK11* co-mutation, and *TSC1/2* alterations were associated with poorer PFS (**Supplementary Table 4**). On multivariable analysis, only poorly differentiated or squamous histology (HR: 2.22; 95% CI: 1.27-3.88) and *STK11* alterations (HR: 3.68; 95% CI: 1.88-7.22) remained independently significant. While *KRAS* subtype not statistically significant, *KRAS* G12D (HR: 2.31; 95% CI: 1.14-4.71) tended to confer poorer PFS (**Table 4**).

**Table 4:** Multivariable models for PFS and OS in the PI Cohort.

Covariate	Level	Progression-free survival				Overall survival			
		Hazard ratio	95% CI		P-value	Hazard ratio	95% CI		P-value
Adenocarcinoma	No	2.22	1.27	3.88	**<.01**				
	Yes	Ref	-	-					
PD-L1 TPS	0%	1.60	0.88	2.94	0.12				
	1-100%	Ref	-	-					
*STK11*	Yes	3.68	1.88	7.22	**<.01**	2.45	1.27	4.70	**<.01**
	No	Ref	-	-		Ref	-	-	
*TERT*	Yes					3.26	1.40	7.61	**<.01**
	No					Ref	-	-	
*TSC1/2*	Yes	1.99	0.80	4.96	1.99	4.57	1.76	11.87	**<.01**
	No	Ref	-	-		Ref	-	-	
*KRAS* Subtype	G12D	2.31	1.14	4.71	0.07	2.58	1.21	5.51	0.06
	G12A/F/R/S	1.07	0.47	2.42		1.85	0.81	4.26	
	G13X	1.96	0.80	4.85		1.86	0.66	5.23	
	G12C/G12V/non-G12/G13	Ref	-	-		Ref	-	-	

p-values in bold indicate statistical significance.

The median OS was 20.0 months (95% CI: 13.2-24.0 months). The 12-month OS rates for *KRAS* G12C, G12V, G12D, G12A/F/R/S, G13X and non-G12/G13 were 70% (95% CI: 53-82%), 68% (95% CI: 41-84%), 50% (95% CI: 21-74%), 61% (95% CI: 29-82%), 27% (95% CI: 2-66%) and 57% (95 CI: 17-84%) respectively (**Figure 2D**, **Supplementary Table 1**). On univariate analysis, ECOG ≥ 2, poorly differentiated or squamous histology, bone and soft-tissue metastases, and alterations in *STK11*, *TERT*, and *TSC1/2* adversely affected OS (**Supplementary Table 4**). On multivariable analysis, histology and sites of metastatic disease were excluded due to the possible interaction with genetic background of the disease and *KRAS* subtypes were included instead. Presence of *STK11* (HR: 2.45; 95% CI: 1.27-4.70), *TERT* (HR: 3.26; 95% CI: 1.40-7.61), and *TSC1/2* (HR: 4.57; 95% CI: 1.76-11.87) remained independently associated with inferior survival. *KRAS* subtype was not significant but *KRAS* G12D (HR: 2.58; 95% CI: 1.21-5.51) tended to confer the worst OS, followed by KRAS G13X (HR:1.86; 95 CI: 0.7-5.2).

## 4 Discussion

*KRAS* is the most mutated oncogene in NSCLC and encompasses a biologically diverse array of variants with differing molecular and clinical behavior. While G12X mutations have garnered significant therapeutic interest, the biology and clinical implications of *KRAS* G13X remain poorly characterized. Building on prior studies describing *KRAS* allele heterogeneity (10–14) and a recent outcome analysis (16), we evaluated the incidence, co-mutation landscape, and clinical outcomes of *KRAS* G13X in a single-institution cohort.

*KRAS* G13X comprised approximately 10% of all *KRAS*-mutated NSCLC in both the CI and PI cohorts consistent with prior reports (10). Patients were slightly younger, more often female, and predominantly former or current smokers. In the PI cohort, *KRAS* G13X tumors demonstrated a distinct co-mutation profile characterized by enrichment of *STK11*, *KEAP1*, *NFE2L2*, and *TSC* genes integral to cellular metabolism, oxidative-stress regulation, and immune evasion (10, 14, 17). Similar enrichment patterns and diminished immunotherapy benefit among non-G12C alleles, including G13X, were also reported by others (10, 16). This convergence supports the hypothesis that *KRAS* G13X potentially represents a biologically aggressive subset driven more by its co-mutational milieu than by the primary *KRAS* variant alone, but further investigation is needed.

In the CI cohort, *KRAS* G13X patients were more often presented with early-stage disease but were less likely to undergo surgery. Median DFS was 22.3 months with *KRAS* G13X showing the poorest 12-month DFS (41%). *TET* alterations in lung adenocarcinoma are uncommon but often co-occur with *KRAS* alterations and are associated with poor outcomes (18). Co-mutation with *TET2* was associated with worse OS but did not impact disease recurrence. Our findings suggest that in non-metastatic setting, both mutational landscape and clinical factors may impact patient’s outcomes.

In the PI cohort, most patients received first-line chemo-immunotherapy or single agent immunotherapy. Median PFS was 8.2 months with *KRAS* G13X demonstrating the poorest 12-month PFS (17%). *KRAS* subtypes were not independently associated with PFS once co-mutations were included in the model. Alterations in *STK11*, *TERT*, and *TSC1/2* genes regulating metabolic adaptation, telomere maintenance, and mTOR signaling (10, 14, 17, 19, 20), emerged as independently associated with inferior outcomes. Their negative prognostic impact likely outweighs the modest effect of *KRAS* allele differences. Notably, *KEAP1* and *NFE2L2* were not significant predictors in our analysis. Trends toward poorer OS for *KRAS* G12D and G13X mirror prior observations (11, 13, 16).

Overall, our data supports that *KRAS* G13X NSCLC may represent a relatively distinct molecular subset defined more by its co-mutation architecture than by the primary *KRAS* variant. The recurrent co-occurrence of *STK11*, *KEAP1*, *NFE2L2*, and *TSC* alterations suggests an immunologically “cold,” metabolically reprogrammed phenotype potentially influencing patient’s outcomes (10, 14, 16). This underscores the need to look beyond allele-centric prognostic and therapeutic frameworks. Rational combination strategies targeting *KRAS* signaling, along with influencing either metabolic or immunologic milieu might be more relevant in *KRAS* G13X tumors (11, 13).

This study is limited by its retrospective design, small sample size, and heterogeneity of treatments over the long inclusion period. However, reproducible molecular patterns across cohorts and alignment with other reports (10, 16) strengthen the biological plausibility of our findings.

## 5 Conclusions

In summary, *KRAS* G13X mutations define a small but clinically relevant subset of NSCLC characterized by frequent *STK11*, *KEAP1*, and *NFE2L2* co-mutations and were associated with poorer outcomes in this study. *KRAS* subtype alone did not seem to be prognostic. Co-mutational landscape appeared central to disease behavior and treatment response. Prospective, multi-institutional studies with larger sample size are warranted to validate these findings and guide the development of tailored therapeutic approaches for *KRAS* G13X NSCLC.

## Data Availability

The original contributions presented in the study are included in the article/**Supplementary Material**. Further inquiries can be directed to the corresponding author.
