# Supplementary material for: Clinical and genomic characterization of KRAS G13X non-small cell lung carcinoma
Source: Front Oncol. 2026 Sep 3;16:1825959. doi: 10.3389/fonc.2026.1825959 (PMC13581628; doi:10.3389/fonc.2026.1825959)
Supplement: Supplementary file 1 [file Table1.docx]

**Title: Clinical and Genomic Characterization of KRAS G13X NSCLC**

**Supplementary Data**

**Table S1:** 12-months Disease-Free, Progression-Free and Overall Survival by KRAS variants (page 2).

**Table S2:** Univariate Analysis for Disease-Free Survival of the CI Cohort (page 3-5)

**Table S3:** Univariate Analysis for Overall Survival of the CI Cohort (page 6-8)

**Table S4:** Univariate Analysis for Progression-Free Survival of the PI Cohort (page 9-11)

**Table S5:** Univariate Analysis for Overall Survival of the PI Cohort (page 12-14)

**Table S1:** 12-months Disease-Free, Progression-Free and Overall Survival by KRAS variants for CI and PI cohorts.

| **KRAS Subtypes** | **CI Cohort** | | **PI Cohort** | |
| --- | --- | --- | --- | --- |
|  | **12-month DFS (95% CI)** | **12-month OS (95% CI)** | **12-month PFS (95% CI)** | **12-month OS (95% CI)** |
| G12C | 80% (63-89%) | 92% (78-97%) | 43% (26-59%) | 70% (53-82%) |
| G12V | 67% (43-83%) | 91% (68-98%) | 33% (13-55%) | 68% (41-84%) |
| G12D | 76% (52-89%) | 90% (67-98%) | 33% (10-59%) | 50% (21-74%) |
| G12A/F/R/S | 80% (20-97%) | 83% (27-97%) | 37% (12-62%) | 61% (29-82%) |
| G13X | 41% (10-70%) | 88% (39-98%) | 17% (1-50%) | 27% (2-66%) |
| Non-G12/G13 | 71% (26-92%) | 100% | 29% (4-61%) | 57% (17-84%) |

**Table S2:** Univariate Analysis for Disease-Free Survival of the CI Cohort

|  | | | **Disease-Free Survival** | | | |
| --- | --- | --- | --- | --- | --- | --- |
|  | | | **-----------------------------------------------------** | | | |
| **Covariate** | **Level** | **N** | **Hazard Ratio** | **95% CI** | | **P-value** |
| Sex | Male | 42 | 1.00 | 0.61 | 1.64 | 0.99 |
|  | Female | 66 | Ref | - | - |  |
| ECOG | 2 | 13 | 2.28 | 1.21 | 4.29 | **0.01** |
|  | 0-1 | 95 | Ref | - | - |  |
| PD-L1 TPS | 0% | 47 | 0.59 | 0.35 | 0.98 | **0.04** |
|  | 1-100% | 60 | Ref | - | - |  |
| Adenocarcinoma | No | 19 | 0.89 | 0.46 | 1.71 | 0.72 |
|  | Yes | 88 | Ref | - | - |  |
| Stage | III | 39 | 1.70 | 1.03 | 2.81 | **0.04** |
|  | I-II | 69 | Ref | - | - |  |
| Surgery | Yes | 61 | 0.57 | 0.35 | 0.94 | **0.03** |
|  | No | 47 | Ref | - | - |  |
| Chemotherapy | Yes | 37 | 1.52 | 0.91 | 2.53 | 0.11 |
|  | No | 71 | Ref | - | - |  |
| Radiation | Yes | 49 | 1.69 | 1.03 | 2.78 | **0.04** |
|  | No | 59 | Ref | - | - |  |
| KRAS Subtype | G12V | 22 | 1.46 | 0.75 | 2.85 | 0.23 |
|  | G12D | 22 | 0.88 | 0.46 | 1.69 |  |
|  | G12A/F/R/S | 6 | 0.80 | 0.19 | 3.42 |  |
|  | G13X | 10 | 2.60 | 1.10 | 6.15 |  |
|  | Non-G12/G13 | 8 | 0.98 | 0.34 | 2.83 |  |
|  | G12C | 40 | Ref | - | - |  |
| KRAS Subtype | G12D | 22 | 0.80 | 0.43 | 1.46 | 0.14 |
|  | G13X | 10 | 2.33 | 1.03 | 5.27 |  |
|  | Non-G12/G13 | 8 | 0.88 | 0.31 | 2.47 |  |
|  | G12A/C/F/R/S/V | 68 | Ref | - | - |  |
| KRAS Subtype | G12D | 22 | 0.79 | 0.44 | 1.45 | 0.13 |
|  | G12A/F/R/S | 6 | 0.71 | 0.17 | 2.96 |  |
|  | G13X | 10 | 2.31 | 1.02 | 5.22 |  |
|  | G12C/G12V/non-G12/G13 | 70 | Ref | - | - |  |
| *TP53* | Yes | 45 | 0.88 | 0.54 | 1.45 | 0.62 |
|  | No | 63 | Ref | - | - |  |
| *ATM* | Yes | 28 | 0.54 | 0.30 | 0.96 | **0.04** |
|  | No | 80 | Ref | - | - |  |
| *STK11* | Yes | 27 | 0.64 | 0.35 | 1.19 | 0.16 |
|  | No | 81 | Ref | - | - |  |
| *NOTCH1/2/3* | Yes | 22 | 1.19 | 0.68 | 2.08 | 0.54 |
|  | No | 86 | Ref | - | - |  |
| *KMT2D* | Yes | 18 | 0.75 | 0.37 | 1.51 | 0.42 |
|  | No | 90 | Ref | - | - |  |
| *NF1/2* | Yes | 14 | 0.98 | 0.48 | 1.98 | 0.95 |
|  | No | 94 | Ref | - | - |  |
| *BRCA1/2* | Yes | 14 | 0.84 | 0.40 | 1.76 | 0.64 |
|  | No | 94 | Ref | - | - |  |
| *FLT1/3* | Yes | 15 | 0.88 | 0.42 | 1.87 | 0.75 |
|  | No | 93 | Ref | - | - |  |
| *SMARCA4/B1* | Yes | 13 | 1.15 | 0.58 | 2.27 | 0.69 |
|  | No | 95 | Ref | - | - |  |
| *ARID1A* | Yes | 12 | 0.46 | 0.21 | 1.03 | 0.06 |
|  | No | 96 | Ref | - | - |  |
| *CDKN1A/1B/2A* | Yes | 14 | 0.88 | 0.43 | 1.79 | 0.71 |
|  | No | 94 | Ref | - | - |  |
| *APC* | Yes | 11 | 0.93 | 0.42 | 2.04 | 0.85 |
|  | No | 97 | Ref | - | - |  |
| *EPHA3* | Yes | 12 | 0.96 | 0.47 | 1.95 | 0.91 |
|  | No | 96 | Ref | - | - |  |
| *NFE2L2/KEAP1* | Yes | 11 | 0.76 | 0.32 | 1.81 | 0.54 |
|  | No | 97 | Ref | - | - |  |
| *ATRX* | Yes | 11 | 1.23 | 0.53 | 2.86 | 0.63 |
|  | No | 97 | Ref | - | - |  |
| *PDGFRA/B* | Yes | 10 | 0.45 | 0.16 | 1.24 | 0.12 |
|  | No | 98 | Ref | - | - |  |
| *SETBP1* | Yes | 11 | 0.68 | 0.31 | 1.49 | 0.34 |
|  | No | 97 | Ref | - | - |  |
| *DNMT3A* | Yes | 10 | 0.94 | 0.43 | 2.06 | 0.87 |
|  | No | 98 | Ref | - | - |  |
| *NTRK1/2/3* | Yes | 10 | 0.14 | 0.02 | 1.01 | 0.05 |
|  | No | 98 | Ref | - | - |  |
| *SETD2* | Yes | 10 | 1.08 | 0.46 | 2.52 | 0.87 |
|  | No | 98 | Ref | - | - |  |
| *TSC1/2* | Yes | 10 | 0.55 | 0.22 | 1.39 | 0.21 |
|  | No | 98 | Ref | - | - |  |
| *CREBBP* | Yes | 8 | 0.76 | 0.32 | 1.78 | 0.52 |
|  | No | 100 | Ref | - | - |  |
| *ATR* | Yes | 6 | 0.80 | 0.25 | 2.57 | 0.71 |
|  | No | 102 | Ref | - | - |  |
| *DOT1L* | Yes | 7 | 1.80 | 0.71 | 4.54 | 0.21 |
|  | No | 101 | Ref | - | - |  |
| *FANCL* | Yes | 7 | 0.58 | 0.18 | 1.85 | 0.36 |
|  | No | 101 | Ref | - | - |  |
| *MSH2/6* | Yes | 7 | 1.57 | 0.68 | 3.66 | 0.29 |
|  | No | 101 | Ref | - | - |  |
| *POLE* | Yes | 7 | 0.64 | 0.23 | 1.83 | 0.41 |
|  | No | 101 | Ref | - | - |  |
| *RB1* | Yes | 7 | 1.02 | 0.37 | 2.81 | 0.98 |
|  | No | 101 | Ref | - | - |  |
| *SLIT2* | Yes | 7 | 1.50 | 0.60 | 3.75 | 0.39 |
|  | No | 101 | Ref | - | - |  |
| *SLX3/4* | Yes | 7 | 0.72 | 0.26 | 2.00 | 0.53 |
|  | No | 101 | Ref | - | - |  |
| *TERT* | Yes | 6 | 0.62 | 0.24 | 1.59 | 0.32 |
|  | No | 102 | Ref | - | - |  |
| *BRAF* | Yes | 6 | 1.22 | 0.48 | 3.05 | 0.68 |
|  | No | 102 | Ref | - | - |  |
| *ERBB2/3/4* | Yes | 6 | 0.56 | 0.14 | 2.29 | 0.42 |
|  | No | 102 | Ref | - | - |  |
| *FGFR1/2/3/4* | Yes | 5 | 0.37 | 0.09 | 1.54 | 0.17 |
|  | No | 103 | Ref | - | - |  |
| *TET2* | Yes | 6 | 1.42 | 0.51 | 3.93 | 0.50 |
|  | No | 102 | Ref | - | - |  |
| *U2AF1/2* | Yes | 6 | 0.66 | 0.20 | 2.10 | 0.48 |
|  | No | 102 | Ref | - | - |  |
| *ZBTB7A* | Yes | 6 | 0.96 | 0.30 | 3.07 | 0.94 |
|  | No | 102 | Ref | - | - |  |
| Age at Diagnosis | Units=1 | 108 | 1.02 | 0.99 | 1.04 | 0.27 |
| Months From Diagnosis to Treatment | Units=1 | 108 | 1.24 | 1.06 | 1.45 | **<.01** |

**Table S3:** Univariate Analysis for Overall Survival of the CI Cohort

|  | | | **Overall Survival** | | | |
| --- | --- | --- | --- | --- | --- | --- |
|  | | | **-----------------------------------------------------** | | | |
| **Covariate** | **Level** | **N** | **Hazard Ratio** | **95% CI** | | **P-value** |
| Sex | Male | 42 | 1.70 | 0.90 | 3.22 | 0.10 |
|  | Female | 66 | Ref | - | - |  |
| ECOG | 2 | 13 | 4.36 | 2.07 | 9.19 | **<.01** |
|  | 0-1 | 95 | Ref | - | - |  |
| PD-L1 TPS | 0% | 47 | 0.64 | 0.33 | 1.25 | 0.19 |
|  | 1-100% | 60 | Ref | - | - |  |
| Adenocarcinoma | No | 19 | 1.08 | 0.47 | 2.49 | 0.85 |
|  | Yes | 88 | Ref | - | - |  |
| Stage | III | 39 | 1.22 | 0.64 | 2.35 | 0.54 |
|  | I-II | 69 | Ref | - | - |  |
| Surgery | Yes | 61 | 0.46 | 0.24 | 0.88 | **0.02** |
|  | No | 47 | Ref | - | - |  |
| Chemotherapy | Yes | 37 | 1.04 | 0.53 | 2.03 | 0.91 |
|  | No | 71 | Ref | - | - |  |
| Radiation | Yes | 49 | 2.29 | 1.18 | 4.46 | **0.01** |
|  | No | 59 | Ref | - | - |  |
| KRAS Subtype | G12V | 22 | 2.80 | 1.16 | 6.76 | 0.35 |
|  | G12D | 22 | 1.69 | 0.71 | 3.99 |  |
|  | G12A/F/R/S | 6 | 1.34 | 0.17 | 10.60 |  |
|  | G13X | 10 | 2.20 | 0.59 | 8.12 |  |
|  | Non-G12/G13 | 8 | 2.12 | 0.57 | 7.85 |  |
|  | G12C | 40 | Ref | - | - |  |
| KRAS Subtype | G12D | 22 | 1.15 | 0.55 | 2.40 | 0.90 |
|  | G13X | 10 | 1.43 | 0.42 | 4.82 |  |
|  | Non-G12/G13 | 8 | 1.38 | 0.41 | 4.65 |  |
|  | G12A/C/F/R/S/V | 68 | Ref | - | - |  |
| KRAS Subtype | G12D | 22 | 1.11 | 0.54 | 2.28 | 0.95 |
|  | G12A/F/R/S | 6 | 0.82 | 0.11 | 6.10 |  |
|  | G13X | 10 | 1.37 | 0.41 | 4.58 |  |
|  | G12C/G12V/non-G12/G13 | 70 | Ref | - | - |  |
| *TP53* | Yes | 45 | 1.29 | 0.68 | 2.44 | 0.43 |
|  | No | 63 | Ref | - | - |  |
| *ATM* | Yes | 28 | 0.58 | 0.27 | 1.27 | 0.17 |
|  | No | 80 | Ref | - | - |  |
| *STK11* | Yes | 27 | 0.59 | 0.26 | 1.35 | 0.21 |
|  | No | 81 | Ref | - | - |  |
| *NOTCH1/2/3* | Yes | 22 | 1.45 | 0.72 | 2.91 | 0.30 |
|  | No | 86 | Ref | - | - |  |
| *KMT2D* | Yes | 18 | 1.07 | 0.44 | 2.57 | 0.89 |
|  | No | 90 | Ref | - | - |  |
| *NF1/2* | Yes | 14 | 0.85 | 0.33 | 2.18 | 0.73 |
|  | No | 94 | Ref | - | - |  |
| *BRCA1/2* | Yes | 14 | 1.63 | 0.71 | 3.74 | 0.24 |
|  | No | 94 | Ref | - | - |  |
| *FLT1/3* | Yes | 15 | 1.20 | 0.49 | 2.92 | 0.69 |
|  | No | 93 | Ref | - | - |  |
| *SMARCA4/B1* | Yes | 13 | 0.94 | 0.36 | 2.43 | 0.90 |
|  | No | 95 | Ref | - | - |  |
| *ARID1A* | Yes | 12 | 0.51 | 0.16 | 1.67 | 0.26 |
|  | No | 96 | Ref | - | - |  |
| *CDKN1A/1B/2A* | Yes | 14 | 1.27 | 0.55 | 2.90 | 0.57 |
|  | No | 94 | Ref | - | - |  |
| *APC* | Yes | 11 | 0.23 | 0.03 | 1.67 | 0.15 |
|  | No | 97 | Ref | - | - |  |
| *EPHA3* | Yes | 12 | 0.89 | 0.31 | 2.52 | 0.82 |
|  | No | 96 | Ref | - | - |  |
| *NFE2L2/KEAP1* | Yes | 11 | 0.61 | 0.18 | 2.05 | 0.42 |
|  | No | 97 | Ref | - | - |  |
| *ATRX* | Yes | 11 | 1.43 | 0.50 | 4.07 | 0.50 |
|  | No | 97 | Ref | - | - |  |
| *PDGFRA/B* | Yes | 10 | 0.49 | 0.12 | 2.04 | 0.33 |
|  | No | 98 | Ref | - | - |  |
| *SETBP1* | Yes | 11 | 0.68 | 0.21 | 2.22 | 0.52 |
|  | No | 97 | Ref | - | - |  |
| *DNMT3A* | Yes | 10 | 0.94 | 0.36 | 2.46 | 0.91 |
|  | No | 98 | Ref | - | - |  |
| *NTRK1/2/3* | Yes | 10 | 0.00 | 0.00 | - | 0.99 |
|  | No | 98 | Ref | - | - |  |
| *SETD2* | Yes | 10 | 1.80 | 0.70 | 4.66 | 0.22 |
|  | No | 98 | Ref | - | - |  |
| *TSC1/2* | Yes | 10 | 1.23 | 0.43 | 3.50 | 0.70 |
|  | No | 98 | Ref | - | - |  |
| *CREBBP* | Yes | 8 | 1.08 | 0.41 | 2.83 | 0.88 |
|  | No | 100 | Ref | - | - |  |
| *ATR* | Yes | 6 | 0.83 | 0.20 | 3.48 | 0.80 |
|  | No | 102 | Ref | - | - |  |
| *DOT1L* | Yes | 7 | 2.03 | 0.71 | 5.78 | 0.19 |
|  | No | 101 | Ref | - | - |  |
| *FANCL* | Yes | 7 | 0.31 | 0.04 | 2.24 | 0.24 |
|  | No | 101 | Ref | - | - |  |
| *MSH2/6* | Yes | 7 | 1.04 | 0.36 | 2.98 | 0.94 |
|  | No | 101 | Ref | - | - |  |
| *POLE* | Yes | 7 | 0.43 | 0.10 | 1.87 | 0.26 |
|  | No | 101 | Ref | - | - |  |
| *RB1* | Yes | 7 | 1.52 | 0.46 | 4.98 | 0.49 |
|  | No | 101 | Ref | - | - |  |
| *SLIT2* | Yes | 7 | 0.39 | 0.05 | 2.84 | 0.35 |
|  | No | 101 | Ref | - | - |  |
| *SLX3/4* | Yes | 7 | 0.25 | 0.03 | 1.85 | 0.18 |
|  | No | 101 | Ref | - | - |  |
| *TERT* | Yes | 6 | 0.75 | 0.22 | 2.55 | 0.64 |
|  | No | 102 | Ref | - | - |  |
| *BRAF* | Yes | 6 | 0.00 | 0.00 | - | 0.99 |
|  | No | 102 | Ref | - | - |  |
| *ERBB2/3/4* | Yes | 6 | 1.23 | 0.29 | 5.17 | 0.78 |
|  | No | 102 | Ref | - | - |  |
| *FGFR1/2/3/4* | Yes | 5 | 0.37 | 0.05 | 2.72 | 0.33 |
|  | No | 103 | Ref | - | - |  |
| *TET2* | Yes | 6 | 3.07 | 1.07 | 8.81 | **0.04** |
|  | No | 102 | Ref | - | - |  |
| *U2AF1/2* | Yes | 6 | 1.62 | 0.49 | 5.34 | 0.43 |
|  | No | 102 | Ref | - | - |  |
| *ZBTB7A* | Yes | 6 | 1.45 | 0.35 | 6.09 | 0.61 |
|  | No | 102 | Ref | - | - |  |
| Age | Units=1 | 108 | 1.02 | 0.98 | 1.05 | 0.43 |
| Months From Diagnosis to Treatment | Units=1 | 108 | 1.03 | 0.83 | 1.27 | 0.81 |

**Table S4:** Univariate Analysis for Progression-Free Survival of the PI Cohort

|  | | | **Progression-Free Survival** | | | |
| --- | --- | --- | --- | --- | --- | --- |
|  | | | **---------------------------------------------** | | | |
| **Covariate** | **Level** | **N** | **Hazard Ratio** | **95% CI** | | **P-value** |
| Sex | Male | 46 | 1.20 | 0.75 | 1.92 | 0.44 |
|  | Female | 54 | Ref | - | - |  |
| ECOG | 2-4 | 17 | 1.36 | 0.77 | 2.42 | 0.29 |
|  | 0-1 | 83 | Ref | - | - |  |
| PD-L1 TPS | 0% | 23 | 1.67 | 0.95 | 2.91 | 0.07 |
|  | 1-100% | 75 | Ref | - | - |  |
| Adenocarcinoma | No | 25 | 2.00 | 1.20 | 3.33 | **<.01** |
|  | Yes | 75 | Ref | - | - |  |
| Group | Recurrent | 35 | 0.94 | 0.58 | 1.53 | 0.80 |
|  | Newly Diagnosed | 65 | Ref | - | - |  |
| Chest: Pleural Effusion / Other Lung / Cardiac | Yes | 58 | 0.74 | 0.46 | 1.19 | 0.21 |
|  | No | 41 | Ref | - | - |  |
| Bone | Yes | 35 | 2.15 | 1.31 | 3.54 | **<.01** |
|  | No | 64 | Ref | - | - |  |
| Brain | Yes | 35 | 0.75 | 0.45 | 1.23 | 0.25 |
|  | No | 64 | Ref | - | - |  |
| Liver | Yes | 7 | 1.97 | 0.90 | 4.34 | 0.09 |
|  | No | 92 | Ref | - | - |  |
| Adrenal | Yes | 12 | 2.26 | 1.19 | 4.30 | **0.01** |
|  | No | 87 | Ref | - | - |  |
| Muscle / Soft Tissue | Yes | 10 | 2.49 | 1.21 | 5.10 | **0.01** |
|  | No | 89 | Ref | - | - |  |
| Initial Treatment for Metastatic Disease | Chemo Only | 13 | 2.00 | 1.01 | 3.96 | 0.07 |
|  | IO Only | 19 | 0.79 | 0.42 | 1.47 |  |
|  | Targeted Therapy | 6 | 0.47 | 0.15 | 1.53 |  |
|  | Chemo + IO | 62 | Ref | - | - |  |
| KRAS Subtype | G12V | 19 | 1.07 | 0.54 | 2.11 | 0.31 |
|  | G12D | 12 | 1.61 | 0.79 | 3.28 |  |
|  | G12A/F/R/S | 14 | 1.20 | 0.56 | 2.59 |  |
|  | G13X | 10 | 2.66 | 1.13 | 6.25 |  |
|  | Non-G12/G13 | 7 | 1.12 | 0.43 | 2.94 |  |
|  | G12C | 38 | Ref | - | - |  |
| KRAS Subtype | G12D | 12 | 1.52 | 0.79 | 2.96 | 0.12 |
|  | G13X | 10 | 2.52 | 1.12 | 5.68 |  |
|  | Non-G12/G13 | 7 | 1.07 | 0.42 | 2.70 |  |
|  | G12A/C/F/R/S/V | 71 | Ref | - | - |  |
| KRAS Subtype | G12D | 12 | 1.56 | 0.80 | 3.04 | 0.12 |
|  | G12A/F/R/S | 14 | 1.16 | 0.56 | 2.41 |  |
|  | G13X | 10 | 2.57 | 1.13 | 5.84 |  |
|  | G12C/G12V/non-G12/G13 | 64 | Ref | - | - |  |
| *TP53* | Yes | 53 | 0.95 | 0.60 | 1.52 | 0.84 |
|  | No | 47 | Ref | - | - |  |
| *STK11* | Yes | 20 | 3.22 | 1.81 | 5.73 | **<.01** |
|  | No | 80 | Ref | - | - |  |
| *ATM* | Yes | 17 | 0.74 | 0.39 | 1.41 | 0.36 |
|  | No | 83 | Ref | - | - |  |
| *NOTCH1/2/3* | Yes | 16 | 1.02 | 0.55 | 1.87 | 0.95 |
|  | No | 84 | Ref | - | - |  |
| *DNMT3A* | Yes | 14 | 0.85 | 0.43 | 1.68 | 0.64 |
|  | No | 86 | Ref | - | - |  |
| *CDKN1A/1B/2A* | Yes | 13 | 0.87 | 0.41 | 1.81 | 0.71 |
|  | No | 87 | Ref | - | - |  |
| *EPHA3* | Yes | 12 | 1.20 | 0.61 | 2.36 | 0.60 |
|  | No | 88 | Ref | - | - |  |
| *BRCA1/2* | Yes | 11 | 0.84 | 0.42 | 1.70 | 0.63 |
|  | No | 89 | Ref | - | - |  |
| *KMT2D* | Yes | 11 | 1.12 | 0.53 | 2.34 | 0.77 |
|  | No | 89 | Ref | - | - |  |
| *NFE2L2/KEAP1* | Yes | 12 | 1.96 | 0.96 | 3.99 | 0.06 |
|  | No | 88 | Ref | - | - |  |
| *SMARCA4/B1* | Yes | 11 | 0.72 | 0.33 | 1.60 | 0.42 |
|  | No | 89 | Ref | - | - |  |
| *ARID1A* | Yes | 11 | 0.88 | 0.40 | 1.93 | 0.75 |
|  | No | 89 | Ref | - | - |  |
| *NF1/2* | Yes | 11 | 1.11 | 0.55 | 2.26 | 0.77 |
|  | No | 89 | Ref | - | - |  |
| *CREBBP* | Yes | 10 | 1.00 | 0.46 | 2.20 | 0.99 |
|  | No | 90 | Ref | - | - |  |
| *FLT1/3* | Yes | 9 | 1.10 | 0.50 | 2.41 | 0.81 |
|  | No | 91 | Ref | - | - |  |
| *MSH2/6* | Yes | 10 | 0.72 | 0.33 | 1.58 | 0.41 |
|  | No | 90 | Ref | - | - |  |
| *POLE* | Yes | 9 | 0.41 | 0.16 | 1.02 | 0.06 |
|  | No | 91 | Ref | - | - |  |
| *SLIT2* | Yes | 9 | 0.59 | 0.24 | 1.48 | 0.26 |
|  | No | 91 | Ref | - | - |  |
| *ATRX* | Yes | 7 | 0.46 | 0.14 | 1.46 | 0.19 |
|  | No | 93 | Ref | - | - |  |
| *SETBP1* | Yes | 8 | 1.40 | 0.64 | 3.08 | 0.40 |
|  | No | 92 | Ref | - | - |  |
| *TERT* | Yes | 8 | 1.98 | 0.90 | 4.35 | 0.09 |
|  | No | 92 | Ref | - | - |  |
| *TSC1/2* | Yes | 8 | 2.66 | 1.21 | 5.86 | **0.02** |
|  | No | 92 | Ref | - | - |  |
| *APC* | Yes | 6 | 0.89 | 0.32 | 2.46 | 0.82 |
|  | No | 94 | Ref | - | - |  |
| *ATR* | Yes | 7 | 0.67 | 0.24 | 1.83 | 0.43 |
|  | No | 93 | Ref | - | - |  |
| *DOT1L* | Yes | 7 | 1.27 | 0.55 | 2.94 | 0.58 |
|  | No | 93 | Ref | - | - |  |
| *NTRK1/2/3* | Yes | 6 | 1.79 | 0.65 | 4.96 | 0.26 |
|  | No | 94 | Ref | - | - |  |
| *PDGFRA/B* | Yes | 6 | 0.51 | 0.16 | 1.64 | 0.26 |
|  | No | 94 | Ref | - | - |  |
| *PTCH1* | Yes | 6 | 0.57 | 0.18 | 1.83 | 0.35 |
|  | No | 94 | Ref | - | - |  |
| *SETD2* | Yes | 6 | 2.25 | 0.96 | 5.31 | 0.06 |
|  | No | 94 | Ref | - | - |  |
| *SLX3/4* | Yes | 5 | 0.20 | 0.03 | 1.42 | 0.11 |
|  | No | 95 | Ref | - | - |  |
| *TET2* | Yes | 6 | 0.71 | 0.25 | 2.00 | 0.52 |
|  | No | 94 | Ref | - | - |  |
| Age at Metastatic Diagnosis | Units=1 | 100 | 1.00 | 0.97 | 1.02 | 0.68 |
| Months From Diagnosis to Treatment | Units=1 | 100 | 0.88 | 0.69 | 1.13 | 0.31 |

**Table S5:** Univariate Analysis for Overall Survival of the PI Cohort

|  | | | **Overall Survival** | | | |
| --- | --- | --- | --- | --- | --- | --- |
|  | | | **---------------------------------------------** | | | |
| **Covariate** | **Level** | **N** | **Hazard Ratio** | **95% CI** | | **P-value** |
| Sex | Male | 46 | 1.53 | 0.91 | 2.59 | 0.11 |
|  | Female | 54 | Ref | - | - |  |
| ECOG | 2-4 | 17 | 2.17 | 1.20 | 3.94 | **0.01** |
|  | 0-1 | 83 | Ref | - | - |  |
| PD-L1 TPS | 0% | 23 | 1.31 | 0.70 | 2.44 | 0.40 |
|  | 1-100% | 75 | Ref | - | - |  |
| Adenocarcinoma | No | 25 | 2.07 | 1.17 | 3.65 | **0.01** |
|  | Yes | 75 | Ref | - | - |  |
| Group | Recurrent | 35 | 0.87 | 0.50 | 1.49 | 0.61 |
|  | Newly Diagnosed | 65 | Ref | - | - |  |
| Chest: Pleural Effusion / Other Lung / Cardiac | Yes | 58 | 0.77 | 0.45 | 1.31 | 0.34 |
|  | No | 41 | Ref | - | - |  |
| Bone | Yes | 35 | 2.03 | 1.17 | 3.52 | **0.01** |
|  | No | 64 | Ref | - | - |  |
| Brain | Yes | 35 | 0.66 | 0.37 | 1.17 | 0.16 |
|  | No | 64 | Ref | - | - |  |
| Liver | Yes | 7 | 1.64 | 0.65 | 4.12 | 0.29 |
|  | No | 92 | Ref | - | - |  |
| Adrenal | Yes | 12 | 1.76 | 0.86 | 3.63 | 0.12 |
|  | No | 87 | Ref | - | - |  |
| Muscle / Soft Tissue | Yes | 10 | 2.99 | 1.44 | 6.18 | **<.01** |
|  | No | 89 | Ref | - | - |  |
| Initial Treatment for Metastatic Disease | Chemo Only | 13 | 1.61 | 0.80 | 3.26 | 0.11 |
|  | IO Only | 19 | 0.75 | 0.37 | 1.51 |  |
|  | Targeted Therapy | 6 | 0.18 | 0.03 | 1.35 |  |
|  | Chemo + IO | 62 | Ref | - | - |  |
| KRAS Subtype | G12V | 19 | 1.43 | 0.68 | 3.04 | 0.22 |
|  | G12D | 12 | 2.34 | 1.07 | 5.13 |  |
|  | G12A/F/R/S | 14 | 1.98 | 0.85 | 4.62 |  |
|  | G13X | 10 | 2.82 | 1.02 | 7.78 |  |
|  | Non-G12/G13 | 7 | 1.51 | 0.56 | 4.10 |  |
|  | G12C | 38 | Ref | - | - |  |
| KRAS Subtype | G12D | 12 | 1.86 | 0.92 | 3.77 | 0.18 |
|  | G13X | 10 | 2.22 | 0.86 | 5.75 |  |
|  | Non-G12/G13 | 7 | 1.22 | 0.48 | 3.11 |  |
|  | G12A/C/F/R/S/V | 71 | Ref | - | - |  |
| KRAS Subtype | G12D | 12 | 1.99 | 0.97 | 4.08 | 0.11 |
|  | G12A/F/R/S | 14 | 1.68 | 0.77 | 3.68 |  |
|  | G13X | 10 | 2.39 | 0.91 | 6.26 |  |
|  | G12C/G12V/non-G12/G13 | 64 | Ref | - | - |  |
| *TP53* | Yes | 53 | 1.06 | 0.63 | 1.78 | 0.83 |
|  | No | 47 | Ref | - | - |  |
| *STK11* | Yes | 20 | 1.95 | 1.08 | 3.54 | **0.03** |
|  | No | 80 | Ref | - | - |  |
| *ATM* | Yes | 17 | 1.02 | 0.51 | 2.01 | 0.97 |
|  | No | 83 | Ref | - | - |  |
| *NOTCH1/2/3* | Yes | 16 | 0.92 | 0.46 | 1.82 | 0.80 |
|  | No | 84 | Ref | - | - |  |
| *DNMT3A* | Yes | 14 | 1.17 | 0.58 | 2.34 | 0.66 |
|  | No | 86 | Ref | - | - |  |
| *CDKN1A/1B/2A* | Yes | 13 | 1.10 | 0.52 | 2.34 | 0.80 |
|  | No | 87 | Ref | - | - |  |
| *EPHA3* | Yes | 12 | 1.38 | 0.67 | 2.82 | 0.38 |
|  | No | 88 | Ref | - | - |  |
| *BRCA1/2* | Yes | 11 | 0.97 | 0.44 | 2.15 | 0.94 |
|  | No | 89 | Ref | - | - |  |
| *KMT2D* | Yes | 11 | 0.95 | 0.38 | 2.38 | 0.91 |
|  | No | 89 | Ref | - | - |  |
| *NFE2L2/KEAP1* | Yes | 12 | 1.27 | 0.57 | 2.81 | 0.56 |
|  | No | 88 | Ref | - | - |  |
| *SMARCA4/B1* | Yes | 11 | 0.90 | 0.39 | 2.10 | 0.81 |
|  | No | 89 | Ref | - | - |  |
| *ARID1A* | Yes | 11 | 0.96 | 0.41 | 2.23 | 0.92 |
|  | No | 89 | Ref | - | - |  |
| *NF1/2* | Yes | 11 | 1.54 | 0.75 | 3.15 | 0.24 |
|  | No | 89 | Ref | - | - |  |
| *CREBBP* | Yes | 10 | 1.58 | 0.71 | 3.50 | 0.26 |
|  | No | 90 | Ref | - | - |  |
| *FLT1/3* | Yes | 9 | 1.39 | 0.60 | 3.27 | 0.44 |
|  | No | 91 | Ref | - | - |  |
| *MSH2/6* | Yes | 10 | 0.97 | 0.44 | 2.15 | 0.95 |
|  | No | 90 | Ref | - | - |  |
| *POLE* | Yes | 9 | 0.46 | 0.17 | 1.28 | 0.14 |
|  | No | 91 | Ref | - | - |  |
| *SLIT2* | Yes | 9 | 0.42 | 0.13 | 1.36 | 0.15 |
|  | No | 91 | Ref | - | - |  |
| *ATRX* | Yes | 7 | 0.39 | 0.09 | 1.59 | 0.19 |
|  | No | 93 | Ref | - | - |  |
| *SETBP1* | Yes | 8 | 1.36 | 0.54 | 3.45 | 0.51 |
|  | No | 92 | Ref | - | - |  |
| *TERT* | Yes | 8 | 2.85 | 1.27 | 6.43 | **0.01** |
|  | No | 92 | Ref | - | - |  |
| *TSC1/2* | Yes | 8 | 3.77 | 1.57 | 9.04 | **<.01** |
|  | No | 92 | Ref | - | - |  |
| *APC* | Yes | 6 | 1.02 | 0.37 | 2.85 | 0.96 |
|  | No | 94 | Ref | - | - |  |
| *ATR* | Yes | 7 | 0.85 | 0.31 | 2.36 | 0.76 |
|  | No | 93 | Ref | - | - |  |
| *DOT1L* | Yes | 7 | 1.68 | 0.72 | 3.93 | 0.23 |
|  | No | 93 | Ref | - | - |  |
| *NTRK1/2/3* | Yes | 6 | 1.54 | 0.56 | 4.28 | 0.41 |
|  | No | 94 | Ref | - | - |  |
| *PDGFRA/B* | Yes | 6 | 0.46 | 0.11 | 1.91 | 0.29 |
|  | No | 94 | Ref | - | - |  |
| *PTCH1* | Yes | 6 | 0.69 | 0.22 | 2.23 | 0.54 |
|  | No | 94 | Ref | - | - |  |
| *SETD2* | Yes | 6 | 1.09 | 0.39 | 3.01 | 0.87 |
|  | No | 94 | Ref | - | - |  |
| *SLX3/4* | Yes | 5 | 0.22 | 0.03 | 1.62 | 0.14 |
|  | No | 95 | Ref | - | - |  |
| *TET2* | Yes | 6 | 0.91 | 0.33 | 2.52 | 0.85 |
|  | No | 94 | Ref | - | - |  |
| Age at Metastatic Diagnosis | Units=1 | 100 | 1.00 | 0.98 | 1.03 | 0.80 |
| Months From Diagnosis to Treatment | Units=1 | 100 | 0.87 | 0.66 | 1.14 | 0.31 |
